# Peer mentoring program through a digital platform for people with systemic sclerosis: A feasibility study

**DOI:** 10.1177/23971983241295911

**Published:** 2024-11-11

**Authors:** Yen T Chen, Nirali Shah, Mary Alore, Sheri Hicks, Nadia Vann, Stephanie Hotz, Adam Pape, Maya Sabbagh, Melissa Cunningham, Dinesh Khanna, Susan L Murphy

**Affiliations:** 1 Department of Physical Medicine and Rehabilitation, University of Michigan, Ann Arbor, MI, USA; 2 Division of Rheumatology, Department of Internal Medicine, University of Michigan, Ann Arbor, MI, USA; 3 University of Michigan Scleroderma Program, Ann Arbor, MI, USA; 4 Office of Patient Experience, University of Michigan, Ann Arbor, MI, USA

**Keywords:** Peer mentoring, social isolation, symptoms, digital platform, systemic sclerosis

## Abstract

**Objective:**

People with systemic sclerosis (SSc or scleroderma), a rare chronic autoimmune disease, often face significant physical and emotional challenges. Peer mentoring, where someone with similar lived experiences offers guidance and support, shows promise in enhancing the well-being of recipients and may benefit individuals with systemic sclerosis. This study aims to evaluate the feasibility and potential health effects of peer mentoring through a digital platform for people with systemic sclerosis.

**Methods:**

We conducted a one-group study to evaluate a 16-week peer mentoring program for people with systemic sclerosis. Mentors and mentees were matched by demographics and systemic sclerosis characteristics. Feasibility was evaluated using Orsmond and Cohn criteria: recruitment, data collection, acceptability, available resources, and participant responses to the program. Perceptions and usability of the peer mentoring program through a digital platform were assessed at week 16 (post-program). The health effects of peer mentoring were measured at baseline, week 8, and week 16.

**Results:**

Five trained mentors and 15 mentees were enrolled. Each mentor was paired with 2–4 mentees. We found that peer mentoring through a digital platform was feasible, acceptable, and had good usability for both mentors and mentees. Mentees reported significantly less anxiety at week 16 (*p* < 0.001). Other improvements in fatigue, pain interference, depressed mood, and resilience were observed, but did not reach statistical significance.

**Conclusion:**

The peer mentoring program through a digital platform was well-received. Results provided preliminary support for the feasibility and potential health benefits of peer mentoring to enhance well-being in people with systemic sclerosis. Findings lay the groundwork for future peer mentoring research in systemic sclerosis.

Systemic sclerosis (SSc) is a rare, complex autoimmune disease characterized by progressive fibrosis of the skin and internal organs.^1^ This disease causes significant symptoms, including fatigue,^2,3^ pain,^3,4^ physical impairment,^3,5^ and psychological distress.^6,7^ Upon diagnosis, reactions range from acceptance and relief to anger and denial.^8,9^ Newly diagnosed individuals often experience anxiety and uncertainty about disease progression and symptom fluctuation, affecting daily activities and social relationships.^10–12^ Work-related concerns, such as job security and potential discrimination, further contribute to their psychological burden.^11–13^ As SSc progresses rapidly^14^ and substantially reduces quality of life,^15,16^ effective disease management becomes crucial. Along with medical treatments, individuals with SSc need guidance and support for self-management.^9,17^ Peer mentoring could provide valuable emotional validation and practical advice.^18–20^

Peer mentoring, involving guidance from individuals with similar health challenges,^18,19^ is valuable for chronic diseases like SSc. It has shown positive effects on coping, anxiety, depressed mood, and social isolation across several rheumatic diseases.^21–23^ Currently, only one evidence-based self-management intervention for SSc offered in a peer mentoring form.^24^ The previous study highlighted that structured health coaching through peer mentoring helps individuals with SSc reduce symptoms and feel more understood and less alone.^25^ Peer support is also important for building resilience,^26^ essential for coping with health-related challenges of living with SSc.^27,28^ However, the multicomponent nature of the prior self-management program makes it difficult to disentangle the specific impact of peer mentoring from other study materials provided.^24^ While peer mentoring has benefited other rheumatic diseases,^21–23^ no study has specifically examined its potential health benefits for individuals with SSc.

Despite its benefits, peer mentoring for people with SSc can be challenging due to physical limitations, geographic barriers, and the disease’s rarity. Digital platforms have recently emerged as effective for delivering peer mentoring,^24,29–31^ offering easy access and flexible scheduling, which are advantageous for people with SSc.^25^ These platforms are particularly feasible during disease flares or outbreaks of communicable diseases like coronavirus disease 2019 (COVID-19) and flu, which pose risks for immunocompromised individuals like those with SSc. Given these considerations, this is the first study to examine the feasibility and potential health benefits of digital peer mentoring for people with SSc. It aims to refine the peer mentoring program to improve well-being and contribute to the limited knowledge on using peer mentoring to enhance health-related outcomes for people with SSc.

## Methods

We conducted a 16-week one-group study to evaluate the peer mentoring program through a digital platform for people with SSc. This study was approved by the University of Michigan Institutional Review Board (HUM00248465).

### Participants

From February 2024 and April 2024, mentors were recruited from the University of Michigan (UM) Scleroderma Peer Mentor Program, and mentees were recruited from an existing SSc registry. Interested individuals were screened by phone for eligibility. People were eligible if they were 18 years or older, had a physician-diagnosed SSc, had access to an Internet-connected device, were able to speak and read English, and were a patient at Michigan Medicine. People were excluded if they had health issues preventing study participation, were in another peer mentoring program, or were undergoing psychological treatments.

### Peer mentoring program

**Figure 1.**
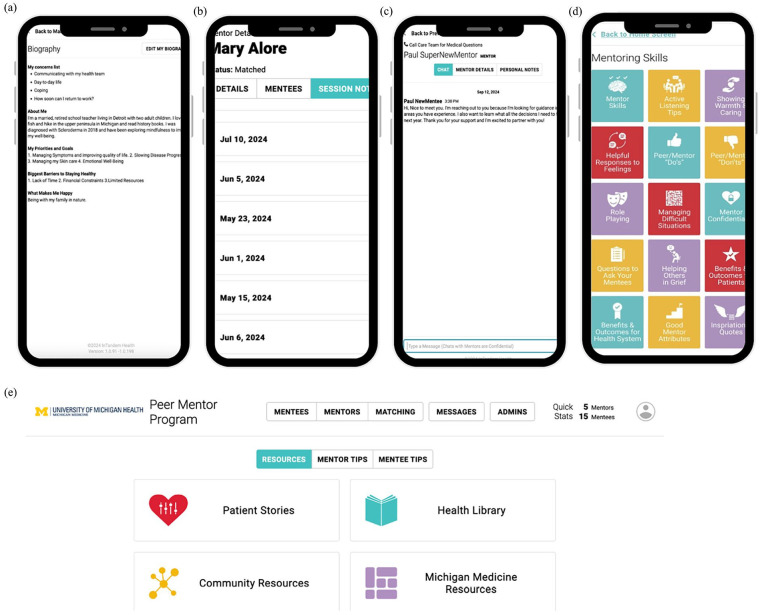
Examples of key features of the peer mentoring digital platform. (a) The platform let mentors and mentees create biographies to introduce themselves. (b) Mentors can use the session notes feature to document their meetings with mentees. The screenshot was shared with the mentor’s consent. (c) Chat function that allows the study team to contact both mentors and mentees if needed. This feature is also available for mentors and mentees to communicate with each other. (d) The resource center from the mentors’ view. Tailored modules are also available for mentees. (e) The peer mentoring digital platform is also accessible through a website that allows the study team to manage all functionalities, including the matching process, contacting mentors or mentees, and reviewing mentors’ session notes.

The first author (Y.T.C.) entered demographics and SSc characteristics of both mentors and mentees into a secure, HIPAA (Health Insurance Portability and Accountability Act)-compliant digital platform. Mentees were matched with mentors using an algorithm based on these characteristics. The platform allowed users to create biographies for introductions. Mentors and mentees were encouraged to connect at least once every month, depending on the mentee’s needs. Interactions could occur through phone calls, texts, or video meetings via the platform. After each interaction, mentors submitted session notes with an option to alert the study team for follow-ups. These notes tracked the frequency, method, and topic of interactions. Participants accessed the peer mentoring program via a website or a supporting app (Figure 1), which included a resource center with tailored modules for both mentors and mentees, personal stories from people with SSc, a health library with self-management information, and other resources provided by Michigan Medicine.

### Outcome measures

*Demographic and SSc characteristics*. Mentors and mentees reported their age, sex, race, education, marital status, and work status. SSc subtype and SSc duration since the date of diagnosis were collected through medical records.

#### Feasibility

Feasibility was evaluated using five guiding questions by Orsmond and Cohn criteria^32^ (Table 1). First, to evaluate recruitment and sample characteristics, we collected data on study flow and recruitment rates (number of consented divided by the number of people approached, ⩾20% was considered a success).^33^ Sample demographic and SSc characteristics for mentors and mentees were examined.

**Table 1.** Feasibility outcomes, guided by Orsmond and Cohns.^32^.

Feasibility evaluation objectives	Outcomes reported
Evaluation of ability to recruit and characteristics of the sample	• Flowchart for mentees • Recruitment rates for both mentors and mentees • Sample characteristics for both mentors and mentees
Evaluation of procedures for data collection	Questionnaire completion rates for both mentors and mentees
Evaluation of acceptability of the program and study procedures	• Dropout rates for both mentors and mentees • Contact frequency and length • Contact methods and topics evaluation • Matching process evaluation • Monitor mentors’ psychological well-being
Evaluation of resources and ability to manage and implement the program	• Study expenses • Evaluation of researcher expertise and skills
Preliminary evaluation of participant responses to the program	• Mentors and mentee’s perceptions of the program (quantitative and qualitative data) • System Usability Scale of the digital platform evaluation for both mentors and mentees

Second, to evaluate data collection procedures, we assessed participants’ compliance with the intended data collection methods. Data were collected through online questionnaires via REDCap, and the completion rate was calculated by dividing the number of completed questionnaires by the total number given to participants.

Third, to evaluate the acceptability of the peer mentoring program and study procedures, we collected data on dropout rates for participants. In the feasibility study, a dropout rate <20% was considered a success.^34^ In addition, we assessed whether the recommended contact frequency (⩾1 contact per month) was met and evaluated the methods and topic of interactions using the mentors’ session notes on the platform. The matching process was also evaluated. Furthermore, to monitor mentors’ well-being and identify any signs of psychological strain related to their mentoring role, we assessed their perceived general self-efficacy using the Patient Reported Outcomes Measurement Information System (PROMIS) General Self-Efficacy short form^35^ at baseline, week 8, and week 16.

Fourth, to evaluate resources and study management, we collected data on the study expenses. The study team also reviewed the required skills and identified the need for the study. Skills not covered by the team were discussed to find solutions.

Fifth, to evaluate participants’ responses to the program, we collected both quantitative and qualitative data. Specifically, perceptions of the peer mentoring program, we collected data using a post-program survey with open-ended questions. In addition, we assessed the usability of the digital platform using the System Usability Scale,^36^ a 10-item questionnaire where responses are rated on a Likert-type scale from 1 (strongly disagree) to 5 (strongly agree). A usability score above 68 is considered above average,^37^ indicating that users agree this digital platform is easy to use and feel confident using it.

#### Health-related outcomes

Mentees completed outcome assessments at baseline, week 8, and week 16. Social isolation was assessed using the PROMIS Social Isolation short form,^38^ which includes four items on a Likert-type scale from 1 (never) to 5 (always). Fatigue and pain interference were assessed with the PROMIS Fatigue and Pain Interference short forms,^39^ each including four items scored from 1 (not at all) to 5 (very much), and both measures are validated in SSc.^40,41^ Anxiety and depressed mood were measured using the PROMIS Anxiety and Depression short forms,^42^ each with four items scored from 1 (never) to 5 (always), also have been validated in SSc.^43^ All PROMIS raw scores were converted to T-scores, where higher scores indicate a greater degree of the domain assessed. Resilience was assessed using the 10-item Connor–Davidson Resilience Scale,^44^ validated in SSc,^45^ and rated on a 5-point Likert-type scale from 0 (not true at all) to 4 (true nearly all the time), with higher scores indicating greater resilience.

### Data analysis

The data analysis plan addressed quantitative feasibility data (e.g., dropout rate and usability score), qualitative acceptability data (i.e., perceptions of the peer mentoring program), and the analysis of data collected using validated measures to assess health-related outcomes. Descriptive statistics are presented as mean and standardized deviation for continuous variables. Categorical variables are presented as numbers and percentages. A repeated-measures analysis of variance (ANOVA) was used to determine the change in each health-related outcome variable (e.g., social isolation and anxiety) over the assessment period. Multiple comparisons between the time points were performed with Bonferroni adjustment (e.g., baseline vs week 8 or baseline vs week 16). The significance of this study was set at *p* < 0.05. All statistical analysis was carried out using IBM SPSS version 27.

## Results

### Evaluation of ability to recruit and characteristics of the sample

**Figure 2.**
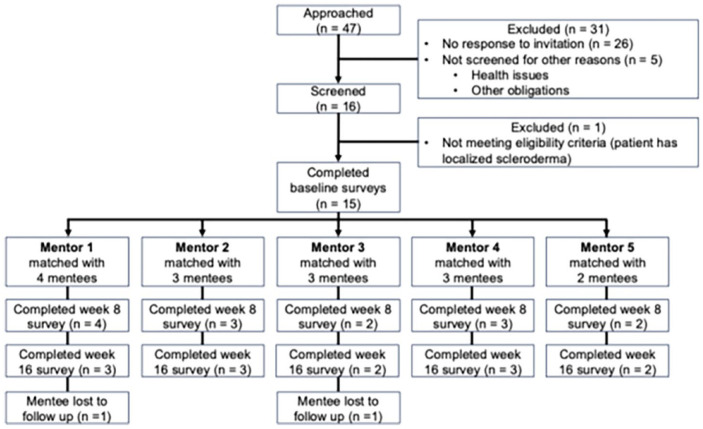
Participant flow diagram for mentees.

Five mentors were initially approached, all of whom were screened, eligible, and agreed to participate in the study. Thus, the recruitment rate for mentors was 100% (five consented of five approached). Figure 2 shows the flowchart of mentees. We approached 47 potential mentees with SSc from the research registry, selecting them based on sign-up order while purposely including a diverse and underrepresentive SSc sample (e.g., males and racial minorities). Of these, 31 were excluded: 26 did not respond, and 5 replied but were excluded due to self-reported health issues. The remaining 16 were screened, with 1 excluded for having localized scleroderma. Therefore, the recruitment rate for mentees was 32% (15 consented of 47 approached).

Table 2 shows participants’ demographic and SSc characteristics. These characteristics align with epidemiological trends for SSc, where SSc is more common in females compared with males.^46^ Although SSc is more frequently diagnosed in Black individuals,^47^ they are often underrepresented in SSc research. The most commonly diagnosed age range for SSc is between the ages of 40 to 50 years,^47^ which is reflected in the age distribution of the mentors and mentees in this study. While the limited subtype is generally more common than the diffuse subtype,^1^ UM Scleroderma Clinic follows and treats more severe cases of SSc, which often include the diffuse subtype. This explains the higher proportion of diffuse subtype cases in our study.

**Table 2.** Sample characteristics of mentors and mentees.

	Mentors (*n* **=** **5)**	Mentees (*n* **=** **15)**
Demographics		
Age, mean (*SD*), years	49.2 (5.2)	56.2 (9.5)
Sex, *n* (%)		
Female	4 (80%)	14 (93%)
Male	1 (20%)	1 (7%)
Race, *n* (%)		
White	4 (80%)	12 (80%)
African American/Asian/others	1 (20%)	3 (20%)
Education status, *n* (%)		
High school/ GED	0	1 (7%)
Some college	0	6 (43%)
College degree	2 (40%)	1 (7%)
Master’s or advanced degree	3 (60%)	6 (43%)
Work status, *n* (%)		
Full-time employed	3 (60%)	5 (33%)
Part-time employed	1 (20%)	1 (7%)
Homemaker	0	2 (13%)
Retired	0	3 (20%)
On disability	1 (20%)	4 (27%)
Marital status, *n* (%)		
Not married	2 (40%)	4 (27%)
Married	3 (60%)	11 (73%)
SSc characteristics		
SSc subtype, *n* (%)		11 (73%)
Diffuse	3 (60%)	4 (27%)
Limited	1 (20%)	0
Overlap	1 (20%)	
Disease duration, mean (*SD*), years		
Disease duration in category	9.2 (5.3)	9.7 (10.0)
0–5 years, *n* (%)	1 (20%)	10 (67%)
>5 years, *n* (%)	4 (80%)	5 (33%)

SSc: systemic sclerosis.

### Evaluation of procedures for data collection

Data were collected using online self-report questionnaires through REDCap. The use of online questionnaires proved feasible. The completion rate for mentors was 100% for all outcome assessments throughout the study. For mentees, the rates were 100% at baseline, 93% at week 8, and 87% at week 16.

### Evaluation of acceptability of the program and study procedures

All mentors remained active throughout the study, with no dropouts. Out of the 15 mentees, 2 dropped out, resulting in a 13% attrition rate. Support needs varied among mentees; of the remaining 13 mentees, 8 (62%) had at least one peer mentoring meeting each month. Qualitative data indicated that missed meetings were mainly due to busy schedules and delayed message alerts, complicating scheduling. Mentors entered 57 session notes on the digital platform, with each contact lasting between 15 min and 1 h. Contacts were primarily via phone (46%), video meetings (33%), or texts (21%). Key discussion topics included symptoms (e.g., fatigue, digital ulcers, and gastrointestinal symptoms), physical activity and diet, disease trajectory, SSc concerns, psychological well-being, treatments, and everyday life.

Regarding the matching process, most mentees had no specific preferences for their mentors. Among 15 cases, 13 mentees were matched with mentors of the same sex and were paired with mentors within a similar age range (±10 years). Eight mentees (53%) were matched with mentors who had the same SSc subtype. To monitor mentors’ well-being and detect psychological strain, we evaluated their general self-efficacy throughout the study. There was no significant change in general self-efficacy or evidence of harm from the mentor role. We also held monthly meetings, sent regular email updates, and followed up via text on the digital platform. All mentors attended at least two of the four monthly meetings. Two mentors reported difficulties contacting their mentees, which were resolved with the first author’s assistance in facilitating communication.

### Evaluation of resources and ability to manage and implement the program

Throughout the feasibility study, we had sufficient resources from UM and did not encounter management difficulties. The digital platform used in this study, typically costing US$250 per month, was provided at no cost for this study. Mentors volunteered their time but were compensated up to US$30 for completing online surveys, as were mentees. All questionnaires were completed online using the University’s free REDCap system. While the current team had the necessary skills, additional expertise from a clinical psychologist and a social worker would be beneficial. This expertise along with collaboration with a statistician would be sought within UM.

### Preliminary evaluation of participant responses to the program

All mentors would recommend digital peer mentoring, noting it as essential support for mentees. Four mentors (80%) valued the platform’s resources, particularly the session notes for easy tracking. However, they reported delays in message alerts and suggested improvements in this feature. All mentees were highly satisfied with the peer mentoring program. In total. 83% reported receiving valuable tips for managing SSc and felt more hopeful: “*It is comforting to have a peer mentor checking in and available to answer questions and discuss my health.*” In total, 75% said peer mentoring improved their diet and exercise routines. In addition, 67% reported reduced anxiety and felt more equipped to advocate for their health. Some mentees suggested adding topics on mental well-being and exercise like hand stretches. They also shared similar concerns as the mentors about delayed message alerts. Finally, the System Usability Scale scores were 70.0 ± 4.9 for mentors and 69.8 ± 24.7 for mentees. Since a score above 68 is considered above average,^37^ these results suggest that both mentors and mentees find the digital platform easy to use and feel confident in using it.

### Potential health effects of peer mentoring

Table 3 shows that a repeated-measures ANOVA revealed a statistically significant difference in mentees’ mean anxiety scores across the time points, F (2, 24) = 11.7, *p* < 0.001. Post hoc analysis with a Bonferroni adjustment showed that the anxiety score significantly decreased from baseline to week 16 (mean difference = 5.5 (95% confidence interval = 2.7–8.2), *p* < 0.001), but there was no significant change from baseline to week 8 or from week 8 to week 16. Although not statistically significant, there were improvements from baseline to week 16 in social isolation, fatigue, pain interference, depressed mood, and resilience.

**Table 3.** Summary of results over time on health-related outcomes.

Variable	Mean (*SD*)			Within group effect *p*	Pairwise comparisons *p*		
	Baseline	Week 8	Week 16		Baseline vs Week 8	Week 8 vs Week 16	Baseline vs Week 16
PROMIS Social isolation, T-score	48.9 (10.1)	48.5 (8.6)	48.7 (8.1)	0.96	>0.99	>0.99	>0.99
PROMIS Fatigue, T-score	56.6 (10.0)	55.6 (10.9)	52.7 (10.9)	0.06	>0.99	0.52	0.06
PROMIS Pain interference, T-score	55.4 (9.7)	53.7 (9.2)	51.2 (8.7)	0.06	0.98	0.44	0.09
PROMIS Anxiety, T-score	54.7 (6.4)	52.8 (7.8)	49.2 (6.6)	<0.001	0.35	0.06	<0.001
PROMIS Depressed mood, T-score	50.1 (7.8)	48.8 (7.6)	45.9 (7.9)	0.08	>0.99	0.43	0.23
Resilience, score	27.4 (6.8)	28.1 (8.1)	29.2 (7.7)	0.12	>0.99	0.42	0.31

*SD*: standard deviation; PROMIS: Patient Reported Outcomes Measurement Information. Analysis using repeated-measures analysis of variance. Pairwise comparisons using Bonferroni corrections.

## Discussion

This is the first study to examine the feasibility and potential health benefits of a digital peer mentoring program for people with SSc. The program was rigorously evaluated using five feasibility criteria by Orsmond and Cohns,^32^ all of which were successfully met, indicating that it is implementable for this patient population. Notably, participants reported reduced anxiety, suggesting the program’s potential as meaningful support and a complementary approach to managing the complex challenges associated with SSc.

Recruitment rates for both mentor and mentee exceeded the 20% threshold. Mentee characteristics matched previous SSc self-management studies,^24,48^ with the representation of males and racial minorities remaining disproportionately low. To address this, future recruitment should target a more diverse sample,^49^ including specific goals and outreach to male and minority SSc support groups. Mentors maintained a 100% questionnaire completion rate, but mentee completion rates gradually decreased, suggesting engagement or burden issues. Periodic reminders and additional support or incentives could help sustain higher completion rates among mentees.

No mentors dropped out of the study. We matched mentees according to their preferences, likely contributing to the high satisfaction and low dropout rate among mentees. However, the amount of peer support that mentees required varied: most mentees required monthly contact and some less frequent, possibly due to differences in SSc duration^25^ or busy schedules. Furthermore, delays in the text alert system also affected scheduling. To improve meeting consistency, addressing the text alert issue and implementing a scheduling tool for coordinating and setting reminders for upcoming meetings should be priorities.

Mentees suggested adding resources on mental well-being and exercise specifically for SSc. To address this, the program could incorporate specialized modules on mental health, hand exercises, and physical activity. In addition, providing mentors with training and resources on these topics could enhance the quality and relevance of the support they offer to mentees. During the study period, mentors were monitored for psychological strain, with no issues detected, aligning with previous SSc study on health coaching through peer mentoring, which reported the mentoring role is safe.^24^

Our findings highlighted the digital platform’s useful features, such as its secure, HIPAA-compliant design, allowing mentors and mentees to communicate without sharing personal contact information. Mentors found session notes helpful for tracking discussions, enhancing continuity and personalized support. Despite issues with delayed text alerts, mentors found the platform easy to navigate and recommended the program, noting its potential to enhance support and foster meaningful connections. Mentees also reported high satisfaction, valuing the personalized support and practical tips for managing SSc. Similar to findings from a previous peer-led program in SSc,^25^ mentees appreciated having a peer mentor to discuss health concerns and benefit from shared experiences. In addition, the System Usability Scale scores were favorable, suggesting that both mentors and mentees found the platform user-friendly and efficient for peer mentoring.

This study explored the potential effects of the peer mentoring program on health-related outcomes. Consistent with previous studies,^21,22^ the program appeared to reduce anxiety but showed no significant effects on other health-related outcomes like fatigue and pain interference. While these preliminary insights suggest potential benefits, the results should be interpreted cautiously, as this study was not powered to detect statistically significant differences.

This study’s limitations include a small sample size and the absence of a control group. However, as a feasibility study, the primary goal was to obtain initial data for planning and implementing a larger-scale study in the future. Consequently, these results are not intended to be generalized to the broader SSc population. Regular monthly peer mentoring was challenging due to technology issues and busy schedules. Future research should implement robust scheduling tools, backup communication channels, automated reminders, and dedicated technical support. In addition, time management training for participants could enhance consistent and effective communication between mentors and mentees.

Overall, this study demonstrates that peer mentoring through a digital platform is feasible, acceptable, and offers potential health benefits for people with SSc. Given the high prevalence of psychological distress and feelings of isolation among individuals with SSc, peer mentoring facilitates social support, which has the potential to alleviate these symptoms and can be a valuable adjunct to standard rheumatologic care. The insights gathered from this study will inform a larger-scale randomized controlled trial to compare the peer mentoring program to a control group, further assessing its benefits for individuals with SSc.

**The statement:** The Editor/Editorial Board Member of JSRD is an author of this article; therefore, the peer review process was managed by alternative members of the Board, and the submitting Editor/Board member had no involvement in the decision-making process.

## References

[1] Denton CP Khanna D. Systemic sclerosis. Lancet 2017; 390: 1685–1699. 28413064 10.1016/S0140-6736(17)30933-9

[2] Willems LM Kwakkenbos L Leite CC , et al. Frequency and impact of disease symptoms experienced by patients with systemic sclerosis from five European countries. Clin Exp Rheumatol 2014; 32(6 Suppl. 86): S88. 25372793

[3] Lescoat A Murphy SL Chen YT , et al. Symptom experience of limited cutaneous systemic sclerosis from the patients’ perspective: a qualitative study. Semin Arthritis Rheum 2022; 52: 151926. 34785028 10.1016/j.semarthrit.2021.11.003 PMC9131352

[4] Lee YC Fox RS Kwakkenbos L , et al. Pain levels and associated factors in the Scleroderma Patient-centered Intervention Network (SPIN) cohort: a multicentre cross-sectional study. Lancet Rheumatol 2021; 3(12): e844–e854. 10.1016/S2665-9913(21)00318-0 38287631

[5] Young A Namas R Dodge C , et al. Hand impairment in systemic sclerosis: various manifestations and currently available treatment. Curr Treatm Opt Rheumatol 2016; 2(3): 252–269. 28018840 10.1007/s40674-016-0052-9 PMC5176259

[6] Jewett LR Razykov I Hudson M , et al. Prevalence of current, 12-month and lifetime major depressive disorder among patients with systemic sclerosis. Rheumatology 2013; 52(4): 669–675. 23256181 10.1093/rheumatology/kes347

[7] Baubet T Ranque B Taïeb O , et al. Mood and anxiety disorders in systemic sclerosis patients. Presse Med 2011; 40(2): e111–e119. 10.1016/j.lpm.2010.09.019 21055901

[8] Nakayama A Tunnicliffe DJ Thakkar V , et al. Patients’ perspectives and experiences living with systemic sclerosis: a systematic review and thematic synthesis of qualitative studies. J Rheumatol 2016; 43(7): 1363–1375. 27134259 10.3899/jrheum.151309

[9] Gumuchian ST Peláez S Delisle VC , et al. Understanding coping strategies among people living with scleroderma: a focus group study. Disabil Rehabil 2018; 40(25): 3012–3021. 28817964 10.1080/09638288.2017.1365954

[10] Khanna D Allanore Y Denton CP , et al. Patient perception of disease burden in diffuse cutaneous systemic sclerosis. J Scleroderma Relat Disord 2020; 5(1): 66–76. 35382406 10.1177/2397198319866615 PMC8922591

[11] Sumpton D Thakkar V O’Neill S , et al. ‘It’s not me, it’s not really me.’ Insights from patients on living with systemic sclerosis: an interview study. Arthritis Care Res 2017; 69: 1733–1742. 10.1002/acr.23207 28129486

[12] Oksel E Gündüzoğlu NÇ. Investigation of life experiences of women with scleroderma. Sex Disabil 2014; 32: 15–21.

[13] Mendelson C Poole JL Allaire S. Experiencing work as a daily challenge: the case of scleroderma. Work 2013; 44(4): 405–413. 22927612 10.3233/WOR-2012-1420

[14] Roofeh D Khanna D. Management of systemic sclerosis: the first five years. Curr Opin Rheumatol 2020; 32(3): 228–237. 32205570 10.1097/BOR.0000000000000711 PMC9161284

[15] Frantz C Avouac J Distler O , et al. Impaired quality of life in systemic sclerosis and patient perception of the disease: a large international survey. Semin Arthritis Rheum 2016; 46(1): 115–123. 27132536 10.1016/j.semarthrit.2016.02.005

[16] van Leeuwen NM Ciaffi J Liem SIE , et al. Health-related quality of life in patients with systemic sclerosis: evolution over time and main determinants. Rheumatology 2021; 60: 3646–3655. 33401302 10.1093/rheumatology/keaa827 PMC8328503

[17] Mouthon L Alami S Boisard A-S , et al. Patients’ views and needs about systemic sclerosis and its management: a qualitative interview study. BMC Musculoskelet Disord 2017; 18: 230. 28558820 10.1186/s12891-017-1603-4 PMC5450385

[18] Dennis CL. Peer support within a health care context: a concept analysis. Int J Nurs Stud 2003; 40(3): 321–332. 12605954 10.1016/s0020-7489(02)00092-5

[19] Doull M O’Connor AM Welch V , et al. Peer support strategies for improving the health and well-being of individuals with chronic diseases. Cochrane Database Syst Rev 2017; 2017: CD005352.

[20] Milette K Thombs BD Dewez S , et al. Scleroderma patient perspectives on social support from close social relationships. Disabil Rehabil 2020; 42(11): 1588–1598. 30761932 10.1080/09638288.2018.1531151

[21] Shadick NA Zibit MJ Iannaccone CK , et al. A development and feasibility study of a peer support telephone program in rheumatoid arthritis. J Clin Rheumatol 2018; 24(6): 346–349. 29389689 10.1097/RHU.0000000000000661 PMC6699178

[22] Sandhu S Veinot P Embuldeniya G , et al. Peer-to-peer mentoring for individuals with early inflammatory arthritis: feasibility pilot. BMJ Open 2013; 3: e002267. 10.1136/bmjopen-2012-002267 PMC3612764 23457326

[23] Lavender EC Anderson AM Dusabe-Richards E , et al. Understanding peer mentorship in supporting self-management of hip and knee osteoarthritis: a qualitative study of mentees’ perspectives. Musculoskeletal Care 2022; 20(1): 180–191. 34314551 10.1002/msc.1580 PMC9290819

[24] Murphy SL Chen YT Alore M , et al. Effects of a resilience-building energy management program on fatigue and other symptoms in systemic sclerosis: a randomized controlled trial. Arthritis Care Res 2024; 76(3): 318–327. 10.1002/acr.25253 PMC10922781 37846437

[25] Chen YT Harper AE Phanhdone T , et al. Impact of a resilience-building energy management intervention for people with systemic sclerosis: a mixed methods study. Rheumatol Adv Pract 2024; 8(2): rkae040. 10.1093/rap/rkae040 PMC11015950 38618141

[26] Mollaei Z Rahemi Z Izadi Avanji FS , et al. The effect of self-care training by peer group on the resilience of patients with cancer: a randomized clinical trial. J Client-centered Nurs Care 2022; 8: 41–50.

[27] Chen YT Hassett AL Khanna D , et al. Resilience partially mediates the association between perceived social isolation and life satisfaction in people with systemic sclerosis. J Scleroderma Relat Disord 2024; 9(2): 154–161. 38910596 10.1177/23971983241232853 PMC11188843

[28] Chen YT Hassett AL Huang S , et al. Peer-led symptom management intervention to enhance resilience in people with systemic sclerosis: mediation analysis from a randomized clinical trial. Arthritis Care Res 2024; 76(9): 1278–1286. 10.1002/acr.25352 PMC11349478 38622109

[29] Colón-Semenza C Latham NK Quintiliani LM , et al. Peer coaching through mhealth targeting physical activity in people with Parkinson disease: feasibility study. JMIR Mhealth Uhealth 2018; 6: e8074. 10.2196/mhealth.8074 PMC5832905 29449201

[30] Lyles CR Sarkar U Patel U , et al. Real-world insights from launching remote peer-to-peer mentoring in a safety net healthcare delivery setting. J Am Med Inform Assoc 2021; 28: 365–370. 33180917 10.1093/jamia/ocaa251 PMC7883966

[31] Allin S Shepherd J Thorson T , et al. Web-based health coaching for spinal cord injury: results from a mixed methods feasibility evaluation. JMIR Rehabil Assist Technol 2020; 7: e16351. 10.2196/16351 PMC7428932 32589148

[32] Orsmond GI Cohn ES. The distinctive features of a feasibility study: objectives and guiding questions. OTJR (Thorofare N J) 2015; 35(3): 169–177. 26594739 10.1177/1539449215578649

[33] Goldstein KM Voils CI Bastian LA , et al. An innovation to expand the reach of peer support: a feasibility and acceptability study. Mil Med 2023; 188: e1569–e1575. 10.1093/milmed/usac295 36226850

[34] Anderson AM Lavender EC Dusabe-Richards E , et al. Peer mentorship to improve self-management of hip and knee osteoarthritis: a randomised feasibility trial. BMJ Open 2021; 11: e045389. 10.1136/bmjopen-2020-045389 PMC8296761 34290063

[35] Salsman JM Schalet BD Merluzzi TV , et al. Calibration and initial validation of a general self-efficacy item bank and short form for the NIH PROMIS. Qual Life Res 2019; 28(9): 2513–2523. 31140041 10.1007/s11136-019-02198-6 PMC6698413

[36] Grier RA Bangor A Kortum P , et al. The system usability scale: beyond standard usability testing. Proc Hum Factors Ergon Soc Annu Meet 2013; 57: 187–191.

[37] Lewis JR . Measuring Perceived usability: SUS, UMUX, and CSUQ ratings for four everyday products. Int J Human–computer Interact 2019; 35: 1404–1419.

[38] Hahn EA Devellis RF Bode RK , et al. Measuring social health in the patient-reported outcomes measurement information system (PROMIS): item bank development and testing. Qual Life Res 2010; 19(7): 1035–1044. 20419503 10.1007/s11136-010-9654-0 PMC3138729

[39] Amtmann D Cook KF Jensen MP , et al. Development of a PROMIS item bank to measure pain interference. Pain 2010; 150: 173–182. 20554116 10.1016/j.pain.2010.04.025 PMC2916053

[40] Hinchcliff ME Beaumont JL Carns MA , et al. Longitudinal evaluation of PROMIS-29 and FACIT-dyspnea short forms in systemic sclerosis. J Rheumatol 2015; 42(1): 64–72. 25362656 10.3899/jrheum.140143 PMC4480645

[41] Morrisroe K Hudson M Baron M , et al. Determinants of health-related quality of life in a multinational systemic sclerosis inception cohort, 2018, https://www.clinexprheumatol.org/abstract.asp?a=12153, https://digital.library.adelaide.edu.Au/dspace/handle/2440/117944 (accessed 31 July 2022). 30183603

[42] Pilkonis PA Yu L Dodds NE , et al. Validation of the depression item bank from the Patient-Reported Outcomes Measurement Information System (PROMIS) in a three-month observational study. J Psychiatr Res 2014; 56: 112–119. 24931848 10.1016/j.jpsychires.2014.05.010 PMC4096965

[43] Morrisroe K Stevens W Huq M , et al. Validity of the PROMIS-29 in a large Australian cohort of patients with systemic sclerosis. J Scleroderma Relat Disord 2017; 2: 188–195.

[44] Campbell-Sills L Stein MB. Psychometric analysis and refinement of the Connor-Davidson Resilience Scale (CD-RISC): validation of a 10-item measure of resilience. J Trauma Stress 2007; 20(6): 1019–1028. 18157881 10.1002/jts.20271

[45] Neyer MA Henry RS Carrier ME , et al. Validity, reliability, and differential item functioning of English and French versions of the 10-item Connor Davidson resilience scale in systemic sclerosis: a scleroderma patient-centered intervention network cohort study. Arthritis Care Res 2023; 75(11): 2369–2378. 10.1002/acr.25139 37128826

[46] Hughes M Pauling JD Armstrong-James L , et al. Gender-related differences in systemic sclerosis. Autoimmun Rev 2020; 19(4): 102494. 32062031 10.1016/j.autrev.2020.102494

[47] Mayes MD Lacey JV Jr Beebe-Dimmer J , et al. Prevalence, incidence, survival, and disease characteristics of systemic sclerosis in a large US population. Arthritis Rheum 2003; 48(8): 2246–2255. 12905479 10.1002/art.11073

[48] Khanna D Serrano J Berrocal VJ , et al. Randomized controlled trial to evaluate an internet-based self-management program in systemic sclerosis. Arthritis Care Res 2019; 71(3): 435–447. 10.1002/acr.23595 PMC6226368 29741230

[49] Stewart AL Nápoles AM Piawah S , et al. Guidelines for evaluating the feasibility of recruitment in pilot studies of diverse populations: an overlooked but important component. Ethn Dis 2020; 30: 745–754. 33250621 10.18865/ed.30.S2.745 PMC7683033

